# Association between Heat-Induced Chemical Markers and Ultra-Processed Foods: A Case Study on Breakfast Cereals

**DOI:** 10.3390/nu12051418

**Published:** 2020-05-14

**Authors:** Francisco J. Morales, Marta Mesías, Cristina Delgado-Andrade

**Affiliations:** Institute of Food Science, Technology and Nutrition (ICTAN-CSIC), Spanish National Research Council, 28040 Madrid, Spain; mmesias@ictan.csic.es (M.M.); cdelgado@ictan.csic.es (C.D.-A.)

**Keywords:** NOVA, NutriScore, acrylamide, hydroxymethylfurfural, breakfast cereals, ultra-processed foods, process-contaminants

## Abstract

Nutritional composition and neo-formed contaminant content in ultra-processed foods, amongst other factors, may contribute to increasing overall risk of non-communicable diseases and cancer. Commercial breakfast cereals (*n* = 53) were classified according to the NOVA approach as un-/minimally processed (NOVA-1, 11%), processed (NOVA-3, 30%), and ultra-processed (NOVA-4, 59%) foods. Acrylamide and hydroxymethylfurfural (HMF) content as heat-induced chemical markers was taken from our research team database. The NutriScore was used as the nutritional profiling system. Samples were distributed between groups A (19%), B (13%), C (38%), and D (30%). No statistically significant differences in acrylamide and HMF were found across the NutriScore groups. Sugar content was the only nutritional descriptor found to be significantly different between processed (11.6 g/100 g) and ultra-processed (23.1 g/100 g) breakfast cereal groups. Sugar content correlated with acrylamide (*p* < 0.001) and HMF (*p* < 0.0001). Acrylamide and HMF contents were not significantly higher in the NOVA-4 group when compared with the NOVA-3 group. However, trends towards higher acrylamide and HMF content are observed, amounting to a change of 75 µg/kg and 13.3 mg/kg in processed breakfast cereals, and 142 µg/kg and 32.1 mg/kg in ultra-processed breakfast cereals, respectively. Thus, the NOVA classification may not reflect the extent of the thermal treatment applied to the breakfast cereal but the type and amount of ingredients incorporated. Ultra-processed breakfast cereal does not predict significantly higher toxicological concern based on acrylamide content than processed breakfast cereals; a clear trend is seen whose contributing factors should be further studied.

## 1. Introduction

The nutritional profile of foods has been gradually modified over the last fifty years due to changes in food systems which alter the availability, accessibility, affordability, and desirability of ready-to-eat foods to consumers. Consequently, dietary patterns within the population have also been modified [1,2]. Home-cooking, which prioritizes minimally processed foods and freshly prepared meals, is pivotal in traditional dietary patterns. Ancestral culinary heritage is being replaced by industrially processed and prepared food products, whose consumption has increased in recent decades [3,4]. However, food processing has played a crucial role in the development of the human condition and its adaptation throughout its 1.7 million-year-old history, since it provides a safe and stable food supply [5,6].

The dietary pattern shifts resulting from the introduction of industrialized processed foods have been accompanied by increases in energy intake, obesity and related chronic non-communicable diseases (NCDs). The most notable diseases include diabetes and cardiovascular and metabolic diseases, which first emerged in high- and middle-income countries [2,7,8,9,10,11]. NCDs represent the main cause of mortality worldwide [12]. Whilst NCDs are multifactorial in nature, nutrition is a common determinant of the progression of NCDs which can be modified through dietary behavior to prevent disease initiation or progression [13]. Industrialized processed foods are associated with a higher intake of simple sugars and saturated fat, and a lower intake of protein and dietary fiber. This leads to poor quality diets [14]. The NOVA system, proposed by Brazilian epidemiologists, is gaining worldwide recognition as a strategy for classifying foods according to their degree of processing and for predicting subsequent risk of NCDs [15]. The NOVA food framework defines processed foods as simple products manufactured from unprocessed or minimally processed foods alongside salt, sugar, oil, or other substances frequently used as culinary ingredients. NOVA introduced the term ultra-processed foods. This aims to identify industrial products formulated with substances derived from food constituents and additives. The final goal of ultra-processed foods is to achieve hyper-palatability or even emulate the characteristics of culinary preparations [16].

Worldwide consumption of ultra-processed foods is increasing significantly. It now represents between 25% and 60% of total daily energy intake in European countries, the US, Canada, New Zealand, and Latin American countries [2,7,17,18]. The SUN prospective cohort study linked a higher consumption of ultra-processed foods (>4 servings daily) with a 62% relative risk increase in all-cause mortality [19]. The Nutrinet-Santé prospective cohort study examined associations between nutrition and health, whilst also studying determinants of dietary behavior and nutritional status within the French population. This large observational study associated a higher consumption of ultra-processed foods with greater risk of suffering cardiovascular, coronary heart and cerebrovascular diseases [20], in addition to greater incidence of cancer in general [21]. The Nutrinet-Santé study concluded that various factors related to processing, the nutritional composition of the final product, additives, contact materials, and neoformed contaminants may play a role in the association between ultra-processed food intake, and overall risk of NCDs and cancer. Specifically, the occurrence of process contaminants such as acrylamide was associated with higher odds of reporting cardiovascular diseases (CVDs) in the National Health and Nutrition Examination Survey (NHANES) study [22].

Process contaminants are defined as compounds produced in a food matrix when it is cooked or processed. They are not present, or are present at much lower concentrations, in the raw or unprocessed food recipe. In particular, heat-induced process contaminants (acrylamide, furan, heterocyclic amines, amongst others) form following the thermal treatment of foods, where the major chemical reaction to take place is the Maillard reaction [23]. The European Food Safety Agency (EFSA) has identified acrylamide content in food as a public health concern due to its relation with the appearance of different types of cancer. This requires continued efforts to reduce consumer exposure to it [24]. Consequently, the European Commission produced the Acrylamide Regulation 2017/2158, compelling food processors and food business operators in Europe to reduce the presence of acrylamide according to the as low as reasonably achievable (ALARA) principle [25]. Another heat-induced chemical in foods is hydroxymethylfurfural (HMF). This forms as an intermediate in both the Maillard reaction and sugar caramelization at high temperatures [26]. HMF is not present in fresh or untreated foods, and rapidly accumulates in proportion to the intensity of heat treatment and storage in carbohydrate-rich foods. HMF is, therefore, a heat-induced chemical marker of the extent of thermal processing, with application in many food categories. Recently, HMF has also been under evaluation as an emerging process contaminant given that a number of studies in murine models have identified its biotransformation into the genotoxic and mutagenic metabolite sulphoxymethylfurfural [27,28].

Breakfast cereals comprise a huge family of cereal-based products. They are obtained through different technological processes which promote the extent of the Maillard reaction, being a relevant source of dietary acrylamide exposure in humans. Thus, the aim of the present study was to investigate the link between the degree of processing as described by the international NOVA food classification system and the occurrence of heat-induced chemical markers in breakfast cereals. In addition, HMF was studied as a heat-induced chemical marker to evaluate the extent of processing, with NutriScore being applied as a reference for overall nutritional quality.

## 2. Materials and Methods

### 2.1. Reagents and Chemicals

Potassium hexacyanoferrate (II) trihydrate (98%, Carrez-I) and zinc acetate dehydrate (>99%, Carrez-II) were obtained from Sigma (St. Louis, USA). 13C3-labelled acrylamide (99% isotopic purity) was obtained from Cambridge Isotope Laboratories (Andover, MA, USA). HMF was purchased from Sigma (St. Louis, MO, USA). Formic acid (98%) was obtained from Panreac (Barcelona, Spain). Deionized water was obtained from a Milli-Q Integral 5 water purification system (Millipore, Billerica, MA, USA). Reversed-phase Oasis-HLB cartridges (30 mg, 1 mL) were provided by Waters (Milford, MA, USA). High performance liquid chromatography (HPLC)-grade methanol was obtained from Merck (Darmstadt, Germany). All other chemicals, solvents and reagents were of analytical grade.

### 2.2. Samples

Fifty-three commercial breakfast cereal samples were purchased from Spanish supermarkets in 2018. Breakfast cereals were identified from product codes, brand names and product names. Breakfast cereals containing dried fruits, nuts, cocoa, filled cereals and novel grains (teff, quinoa, kamut) were intentionally excluded from the survey in order to avoid an unpredictable external occurrence of heat-process contaminants. Breakfast cereals were classified according to the type of the cereal in the formula, with these being rice (*n* = 3), oat (*n* = 6), maize (*n* = 14), wheat (*n* = 14), and multi-cereal (*n* = 16). Samples had different attributes in terms of processing and/or ingredient content. Ingredient content corresponded to muesli (6 samples), puffed/expanded (18 samples) and flakes (29 samples). All brands are well known to consumers since they are widely distributed within all European countries. The survey covered 20 of the principal breakfast cereal manufacturers in Europe. The nutritional composition of samples was recorded from manufacturer declarations and obtained from labels provided on the front of the packaging. The acrylamide and HMF content of samples was obtained from a dataset previously developed by the present research team [29,30].

### 2.3. Processing Classification System of Foods NOVA

Sample classifications based on the degree of processing were stablished using the NOVA system, as described by Monteiro et al. [31]. NOVA establishes four groups which correspond to unprocessed or minimally processed foods (NOVA-1), processed culinary ingredients (NOVA-2), processed foods (NOVA-3) and ultra-processed foods (NOVA-4). Most of the samples in the NOVA-3 group included added sugar, whereas NOVA-4 samples also include ingredients not commonly used in culinary preparations. Breakfast cereals classified as ultra-processed foods have a complex formulation which includes ingredients different to sugar, vitamins, or mineral salts, such as other sweeteners (starch, maltodextrin, dextrose, honey, caramel, extracts of malted and toasted cereals, glucose syrups, inverted sugar, caramel and maltitol), cinnamon, vegetal oil, salt, lactose, skimmed milk powder, and additives (emulsifying, coloring, flavoring, antioxidant, anti-caking, raising agents).

### 2.4. Dietary Nutrient Classification System of Foods NutriScore

The NutriScore scheme was used to classify the nutritional value of samples according to the recommendations of the European Parliament on the provision of nutritional information to consumers [32]. NutriScore is a nutrition label that converts the nutritional value of products into a simple code consisting of 5 letters (A, B, C, D, E), with each corresponding to a color [33]. Breakfast cereals were classified according to the 18th version (1 October 2019) approved by Santé Publique France [34]. Nutritional value is calculated from a formula that considers the nutrients found per 100 g of food. The score for each food is based on a unique, discrete and continuous scale ranging from −15 (most healthy) to +40 (least healthy). The NutriScore computes negative points for energy (0–10 points), saturated fat (0–10 points), total sugar (0–10 points) and sodium (0–10 points), and positive points for fruit and vegetables, legumes and nuts (0–5 points), fiber (0–5 points) and protein (0–5 points). The total score is calculated by subtracting the positive points from the negative ones. This produces a final score, with scores then being categorized to one of five groups (scores of between −15 and −1 are assigned to group A, scores between 0 and 2 to group B, between 3 and 10 to group C, between 11 and 18 to group D, and between 19 and 40 to group E). Next, dietary nutrient profile descriptors were considered; energy density (kcal/100 g), protein (g/100 g), total fat (g/100 g), saturated fat (g/100 g), carbohydrate (g/100 g), sugars (g/100 g), salt (expressed as sodium content, mg/100 g), and dietary fiber (g/100 g).

### 2.5. Determination of Acrylamide

Determination of acrylamide was based on ISO:EN:16618:2015 with minor modifications, using LC-ESI-MS/MS with isotopic dilution. This process was previously described by Mesias et al. [29]. The accuracy of this analytical procedure was evaluated through proficiency tests launched by the Food Analysis Performance Assessment Scheme (FAPAS) program, yielding z-scores between −0.3 and 0.3. Precision (reproducibility) was <10% and recovery was between 84% and 109%. Quantitation and detection limits were set at 20 and 6 μg/kg, respectively. Results were expressed as μg/kg of sample.

### 2.6. Determination of HMF

HMF was determined following aqueous extraction and clarification using HPLC-DAD, as described by Mesias et al. [30]. Precision (reproducibility) was lower than 10% and recovery was between 89% and 105%. Quantitation and detection limits were set at 0.3 and 0.1 mg/kg, respectively. Results were expressed as mg/kg of the sample.

### 2.7. Statistical Analysis

Statistical analyses were performed using Statgraphics Centurion XV (Herndon, VA, USA) and SPSS in its version 23.0 (SPSS Inc., Chicago, IL, USA). In cases where acrylamide or HMF content was lower than the detection limit, the data were recorded as half of this limit. Student t-test and analysis of variance (one-way ANOVA) with Bonferroni’s multiple comparisons post hoc test was used. The homogeneity of variances was determined with Levene’s test and the Shapiro–Wilk W test was applied to check the normality. The association between heat-induced markers (acrylamide and HMF) with nutritional descriptors (energy, sugar, saturated fat, sodium, fiber and protein) was assessed using Pearson’s correlation test and the association with NOVA and NutriScore groups was evaluated by computing Spearman’s rank correlation test. A *p*-value < 0.05 was considered to be statistically significant.

## 3. Results and Discussion

### 3.1. Classification According to NutriScore System

Table 1 summarizes outcomes of the NutriScore nutrient profiling classification system for the entire breakfast cereal dataset. The descriptor with the lowest variability was total energy (7.1% of coefficient of variation, CV), whilst saturated fatty acid content outcomes showed the largest variability (165.0%, CV). Coefficients of variation for protein, sugar, sodium, and fiber content were 35.0%, 78.2%, 89.2%, and 100.8%, respectively. These results are in line with those recently reported by Vermote et al. [35] from a survey carried out in 2018 on breakfast cereals marketed in Belgium. Average and standard deviation relating to NutriScore descriptors were 1685 ± 147 kJ/100 g, 19.1 ± 9.4 g/100 g, 2.5 ± 2.3 g/100 g, 158 ± 158 mg/100 g, 7.2 ± 3.7 g/100 g, and 9.2 ± 2.1 g/100 g for energy (transformed to kJ), sugar, saturated fat, sodium (recalculated from salt), fiber, and protein, respectively. The present study also found the lowest variability to relate to total energy content and highest variability to relate to descriptors of both saturated fat and sodium content.

**Figure 1:**
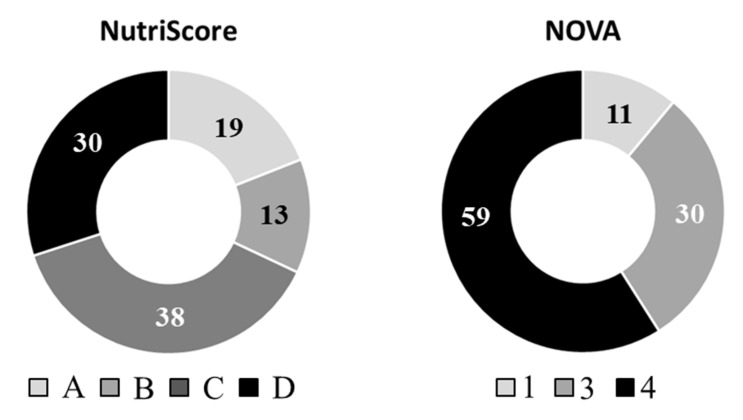
Distribution of breakfast cereals according to NutriScore and NOVA food classification groups. NutriScore groups (A, B, C, D). NOVA groups (1, 3, 4). Numbers refer to percentage of cases.

NutriScore is established as a five-color nutrition front-of-pack labelling system in a growing number of European countries (France, Belgium, Spain, Germany and Switzerland). The NutriScore system includes values which range from −15 to 40 and evaluate overall nutritional food quality. The NutriScore incorporates unfavorable factors (negative points), such as calories, saturated fatty acids, sugars, and sodium, and favorable factors (positive points), such as protein, fiber, fruits and vegetables, legumes and nuts, and olive, nut, and colza oils [36]. Vegetables and fruit content were not considered in the present investigation since samples containing cocoa, dried fruits and nuts were intentionally excluded. Scores pertaining to the breakfast cereals in the present study ranged from −5 to 17 points (data not shown). The sample distribution across NutriScore groups was as follows: 10 samples (19%) with NutriScore A, 7 samples (13%) with NutriScore B, 20 samples (38%) with NutriScore C, 16 samples (30%) with NutriScore D, and zero samples with NutriScore E (Figure 1). Levels of descriptors in each NutriScore group are detailed in the Supplementary Table S1. The distribution is in line with that described by Vermote et al. [35] for NutriScore B and C with 11.5% and 40.6% of breakfast cereals being assigned, respectively. However, percentages for Nutri-Score A, D and C were noticeably different (29.7, 17.9% and 0.3%, respectively).

The role of breakfast cereals in a balanced diet is widely recognized [37,38]. The healthiest breakfast cereals correspond to three samples of wholegrain oat flakes and two samples of wholegrain wheat with a NutriScore value of −5 (group A). The sample with the poorest nutritional quality has a NutriScore value of 17 (group D) and corresponds to a breakfast cereal sample formulated with a mixture of cereals, including oat, wheat and rice. This sample had the highest saturated fat (9.5 g/100 g) and energy (1946 kJ/100 g) content, moderate sugar content (18.4 g/100 g), and low sodium content (116 mg/100 g). Considering the average nutritional profile of the whole dataset for each descriptor, regardless of the sample composition, the average NutriScore classification was B. This is because the outcome of subtracting negative points (10) from positive points (10) of each descriptor was zero, remaining within the range from 0 to 2 (NutriScore B). However, it is more realistic from a technological point of view when considering the average nutritional score obtained between each individual sample since it represents the food as sold. When data were considered in this way, the mean NutriScore value was 6, corresponding to group C (3 to 10 points). Julia et al. [39] investigated the ability of a five-category system for nutritional information (the five-color nutrition label—5-CNL) to discriminate the nutritional quality of breakfast cereals in the French market (including filled cereals, *n* = 380). This classification system was developed prior to the current NutriScore but is based on the same scheme of positive and negative points. NutriScore is derived from the modified Nutrient Profiling System of the British Food Standards Agency (FSAm-NPS) [40]. Color categories were: ‘Green’ (−15 to −2), ‘Yellow’ (−1 to 3), ‘Orange’ (4 to 11), ‘Pink’ (12 to 16) and ‘Red’ (17 and above). The percentage distribution of included breakfast cereals across categories was as follows: “green” (11%), “yellow” (9%), “orange” (59%), “pink” (18%), and “red” (3%). This is in line with the average nutritional score obtained in our dataset for Spanish breakfast cereals according to NutriScore.

### 3.2. Sample Classification According to NOVA System

Figure 1 depicts sample distribution across NOVA food system groups. A total of 6 samples (11%) were identified as unprocessed or minimally processed foods (NOVA-1), 16 samples (30%) as processed foods (NOVA-3), and 31 samples (59%) as ultra-processed foods (NOVA-4). All breakfast cereals included in NOVA-1 were wholegrain, specifically oat flakes (*n* = 5) and multicereal (oat, wheat, rye, and barley) flakes (*n* = 1). To our knowledge, no previous studies in the scientific literature exist that have classified marketed breakfast cereals according NOVA groups. According to Nutrinet-Sante [20], the main food groups contributing to ultra-processed foods intake are sugary products (28%, including confectionaries, ice cream, pastries and sweetened dairy desserts). These are followed by ultra-processed fruit and vegetables (18%, including instant powder dehydrated vegetable soups and broths, vegetable nuggets and fruit-based sweetened desserts), beverages (16%, for example, sodas and sugary and artificially sweetened non-carbonated beverages), starchy foods and breakfast cereals (12%, including pre-packaged bread, industrial dough, ready-to-eat industrial pasta or potato-based dishes, breakfast cereals), and processed meat and fish (11%, for example, nuggets, fish fingers, sausages and processed ham).

Generally speaking, the differences seen between the samples in the NOVA-3 and NOVA-4 groups are related to the amount and type of ingredients and additives, as well as the use of refined foodstuffs or wholegrains. Ultra-processed foods (NOVA-4) are defined as industrial formulations which are manufactured from ingredients extracted or derived from foods (i.e., sugar, plant oils, modified starches) and additives (i.e., colorants, flavorings, emulsifiers, artificial sweeteners). They are formulated through a series of processes to create hyper-palatable, convenient, accessible and attractive products which can be consumed in any place and at any time [9]. NOVA-3 breakfast cereals were formulated with rice (*n* = 1), maize (*n* = 6), wheat (*n* = 3) or a mixture of cereals (*n* = 4) (Supplementary Figure S1), with half of them containing wholegrains (*n* = 8). Samples in NOVA-3 were mostly flakes (*n* = 10), but also puffed cereals (*n* = 3) and muesli (*n* = 3). A common characteristic of NOVA-3 samples is that they contain added sugar and vegetable oil. These are also classical culinary ingredients of the NOVA-2 group; however, those in NOVA-3 contain a very limited number of additives. Breakfast cereals in NOVA-4 were formulated with oat (*n* = 1), maize (*n* = 8), wheat (*n* = 11) or a mixture of cereals (*n* = 11) (Supplementary Figure S1). Most NOVA-4 samples are refined grains (*n* = 19) or a mixture of refined and wholegrain (*n* = 12). Samples in the NOVA-4 group were puffed cereals (*n* = 15), flakes (*n* = 13) and muesli (*n* = 3). The breakfast cereals assigned as NOVA-4 in our dataset not only contain added sugar and vegetable oil, but also maltodextrin, dextrose, caramel, extracts of malted and toasted cereals, glucose syrup or caramel, inverted sugar, maltitol, skimmed milk powder, several additives (emulsifying, coloring, flavoring, antioxidant, anti-caking, raising agents), and vitamins and mineral salts.

Table 2 summarizes the distribution of the nutritional profile of breakfast cereals across the NOVA groups. Samples in NOVA-4 had a sugar and sodium content that was higher than the average content of these nutritional descriptors for the rest of the dataset (Supplementary Figure S2). This nutritional distribution across NOVA system was expected since ultra-processed foods are characterized by their low nutritional quality and high energy density. This is because they contain a higher content of total fat, saturated fat, carbohydrate, sodium and added sugar, alongside a lower content of protein, fiber, vitamins, zinc, potassium, calcium, iron, and fruits and vegetables [31,41,42,43,44,45,46,47].

**Figure 2:**
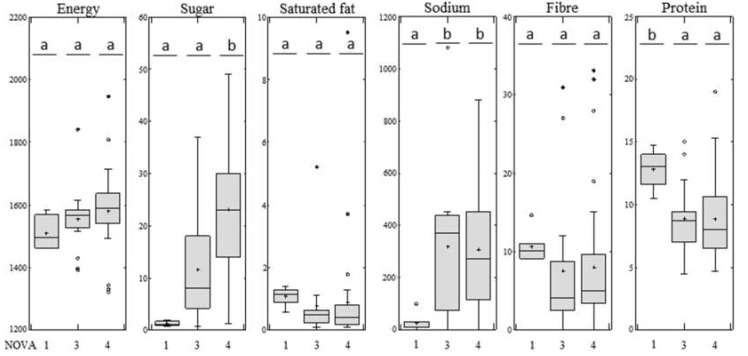
Bar plot showing the distribution of negative (energy, sugar, saturated fat and sodium) and positive (fiber and protein) NutriScore descriptors according to different NOVA groups. Energy (kJ/100 g), sugar (g/100 g), saturated fat (g/100 g), sodium (mg/100 g), fiber (g/100 g) and protein (g/100 g). Different letters indicate significant differences between NOVA categories (ANOVA and Bonferroni post-hoc test, *p* < 0.05). NOVA-1 (unprocessed or minimally processed foods, *n* = 6), NOVA-3 (processed foods, *n* = 16), NOVA-4 (ultra-processed foods, *n* = 31).

The relationship between nutritional quality, as described by the NutriScore, and the NOVA system was further studied. Mean NutriScore ratings for each group of samples were evaluated according to their NOVA categories. The average NutriScore rating in NOVA-1 was −3 (NutriScore A), NOVA-3 produced an average score of 4 (NutriScore C) and NOVA-4 revealed an average rating of 9 (NutriScore C). Figure 2 describes the distribution of the NutriScore descriptors (energy density, sugar, saturated fat, sodium, fiber and protein) across the three NOVA breakfast cereal groups. No statistically significant differences were found in relation to energy density (*p* = 0.3880), saturated fat (*p* = 0.9056) and fiber (*p* = 0.7272). In contrast, differences were statistically significant for sugar content (*p* = 0.0001), sodium (*p* = 0.0280) and protein content (*p* = 0.0179). NOVA-1 showed significantly lower sodium content and higher protein content than NOVA-3 and NOVA-4. Energy density was higher in the group of ultra-processed samples, although differences were not found to be statistically significant from NOVA-3. Ultra-processed breakfast cereals showed a significantly higher sugar content than processed and un/minimally processed samples. The average sugar content was 1.3 (0.8–1.8, 95% C.I.), 11.6 (6.1–7.2, 95% C.I.), and 23.1 g/100 g (18.4–27.7, 95% C.I.) for NOVA-1, NOVA-3 and NOVA-4, respectively. Sugar content was the only statistically different nutritional descriptor between processed and ultra-processed breakfast cereal groups. These results are in line with previous epidemiological studies which found sugar content to be significantly higher in ultra-processed foods than in processed foods [48,49].

A systematic review concluded that the high total sugar intake associated with frequent ready-to-eat cereal consumption is of great concern when considering the health outcomes linked to this food [38]. Besides sugar intake, the high glycemic index (GI) of this food matrix, via puffing, extrusion, popping, etc. leads to food breakdown, which favors the rapid digestion of starch. Increasing the bran levels of breakfast cereals could be a tool to control the GI of this foodstuff. In fact, studies comparing the plasma glucose kinetics of low (bran cereal) and high (corn flakes) GI breakfast cereals have established that the lower GI of bran cereal was not due to a lower rate of glucose appearance. Instead, earlier postprandial hyperinsulinemia and an earlier increase in the rate of glucose disappearance attenuated the increase in the plasma glucose concentration [50]. This is particularly important amongst children and adolescents [51] since they form a specific group which regularly consumes breakfast cereals [52]. Indeed, most of the main breakfast cereal producers market tailored brands for this segment of the population. In the present study, 17 samples were identified to be intended for the consumption of children. It is noteworthy to mention that 88% (*n* = 15) of these samples were classified as ultra-processed (NOVA-4). However, Nutri-Score classified eight samples (47%) as moderate nutritional quality (NutriScore C), whilst nine samples (53%) were regarding as being of poor nutritional quality (NutriScore D). Following NutriScore evaluation, Vermote et al. [35] concluded that breakfast cereals available on the Belgian market were predominantly unhealthy. As a result, they recommended reformulation and urged policy restrictions on marketing approaches and claims, particularly those targeting children.

### 3.3. Heat-Induced Chemical Indexes across NOVA Groups

#### 3.3.1. Heat-Induced Process Contaminant

The acrylamide content of breakfast cereals varied from < LOQ (20 µg/kg) to 382 µg/kg (Table 1). For statistical analysis, 10 µg/kg (half of the quantitation limit for acrylamide) was assigned to two samples which had an acrylamide content < LOQ. These samples were wholegrain oat and multicereal formula, with both being assigned to NOVA-1 and NutriScore-A. The mean acrylamide content was 108 µg/kg and median content was 69 µg/kg (Table 1). The results are in line with the large EFSA survey which covered 75% of the EU market (*n* = 1230) and showed a mean of 113 μg/kg and a median of 67 μg/kg [24]. Six samples (11%) had an acrylamide content higher than 300 µg/kg. Of these, the sample with the highest acrylamide content (382 µg/kg) was a puffed wheat cereal with added honey (NOVA-4, NutriScore-D).

Breakfast cereals contribute to the chronic dietary acrylamide exposure within all age ranges of the population [24] and are thus regulated by the European Commission [25]. Exposure is higher in toddlers and adolescents. Delgado-Andrade et al. [53] estimated that the mean acrylamide exposure of Spanish male adolescents (11–14 years old) was 0.534 μg/kg bw/day, with breakfast having a relevant contribution (32%) to total dietary intake. The category “breakfast cereals” was further classified according to the FoodEx2 food categorization system, used by EFSA [24] and the Commission Regulation 2017/2158 [25]. The European Commission established benchmark levels (BL) for the presence of acrylamide in breakfast cereals and identified three groups: 1) bran products and wholegrain cereals, and gun puffed grains (BL = 300 µg/kg); 2) wheat- and rye-based products (BL = 300 µg/kg), and 3) maize, oat, spelt, barley and rice-based products (BL = 150 µg/kg). In the present dataset, 25 samples corresponded to EU-group-1, 8 samples to EU-group-2, and 20 samples to EU-group-3. Different categories of the “breakfast cereal” group were distributed across the NOVA system (Supplementary Figure S3). It is noteworthy that all samples with acrylamide content higher than BL in each food group all belonged NOVA-4. Of these, two samples corresponded to EU-group-1, four samples to EU-group-2, and one sample to EU-group-3.

#### 3.3.2. Heat-Induced Chemical Marker of the Extent of Processing

HMF is a classical chemical index widely used to evaluate the extent of thermal treatment. It is especially used in milk, dried fruits and cereal-based products [26]. However, given its toxicological properties in murine models, HMF is suspected to have potential genotoxic and mutagenic effects in humans through its metabolism product, sulphoxymethylfurfural [27,54]. It is, therefore considered to be as an emerging process contaminant. The present study considers HMF as a well-known heat-induced chemical index for identifying the extent of thermal processing in foods, particularly in breakfast cereals. The HMF content in the overall dataset of breakfast cereals varied from 0.3 to 159.6 mg/kg, with a mean of 21.8 mg/kg and a median value of 13.5 mg/kg (Table 1). The existing literature suggests the occurrence of HMF in breakfast cereals varies between 0.4 and 194 mg/kg [55,56]. The major route of HMF formation in heat-treated cereal-based foods is through the thermal caramelization of sugars [57]. The degradation of reducing sugars (fructose and glucose) undergoes ß-elimination or dehydration via an enediol intermediate. This forms key α-dicarbonyl intermediates, such as 3-deoxyhexulose, which react to form HMF [58]. The sample with the highest HMF content (159.6 mg/kg) corresponded to refined wheat flakes with the addition of 4% honey. This sample also contained the highest sugar (49 g/100 g) content and was classified as NOVA-4 and NutriScore-D. In contrast, the lowest HMF content (0.3 mg/kg) pertained to six samples of wholegrain oat flakes. All were classified as NOVA-1, with five having NutriScore-A and one NutriScore-B.

### 3.4. Relationship between Heat-Induced Chemicals and Food Classification Systems

Bivariate correlation analysis was applied to investigate in greater detail the strength and direction of relationships between heat-induced chemical indexes, nutritional dietary descriptors and food classification systems (Table 3). Acrylamide content correlated significantly with HMF, sugar content and NOVA food classification system scores. No differences were revealed in relation to energy, saturated fat, sodium, fiber, protein content and NutriScore. HMF content significantly correlated with acrylamide, sugar content and NOVA and NutriScore classifications. No differences were found in relation to energy, saturated fat, sodium, fiber and protein content.

There was a positive and strong correlation between acrylamide and HMF (*p* < 0.0001, r = 0.4987), independent of the type of cereal, type of processing or nutrient content. This relationship was partly expected since both chemicals form during thermal treatment, although the types of precursors, chemistry underlying the reaction and the kinetic behavior are different [57]. Free asparagine and carbonyl compounds are required to form acrylamide from the Maillard reaction [59]. However, sugars alone through thermal caramelization, or in combination with amino acids through the 1,2-enolisation route of the Maillard reaction, contribute to the formation of HMF [26,58].

Regarding sugar content, there was a positive and significant relationship with acrylamide (*p* = 0.0005, r = 0.4648) and HMF (*p* < 0.0000, r = 0.5547) (Table 3). To get more insight into this relationship, the subgroup of breakfast cereals with added honey (*n* = 13) was investigated further. Syrups and caramel also provide a source of HMF and HMF precursors; however, these were not considered since their content was not quantified on the package labelling of the sample. Honey content varied from 0.2% to 5%. All samples with added honey were classified as NOVA-4, and had a NutriScore of C or D. Addition of honey to the breakfast cereals formula contributed to significant increases in HMF (*p* = 0.0047) and acrylamide (*p* = 0.0195) regardless of the NOVA or NutriScore classification systems. The average HMF content in samples without added honey was 16.6 mg/kg (10.6–22.7, 95% C.I.), and was 42 mg/kg (31.4–52.6, 95% C.I.) in samples with added honey. With regard to acrylamide, the average content was 89 µg/kg (67–111, 95% C.I.) and 165 µg/kg (127–204, 95% C.I.) for samples without and with added honey, respectively. In order to avoid bias when correlating heat-induced chemical indexes with sugar content, the breakfast cereal with honey sub-group was removed from the analysis. Nonetheless, a statistically significant correlation was still found between both acrylamide (*p* = 0.0074) and HMF (*p* = 0.0145), and sugar content. This is independent of the type of cereal or food classification system applied. It is known that the intake of food with a higher glycemic index is associated with cardiovascular disorders [60], whilst dysbiosis also increases pro-inflammatory potential in the intestinal bowl [61]. Our results show that the higher occurrence of acrylamide in breakfast cereals with a higher sugar content could contribute to risk factors associated with the overconsumption of sugary foods. Samples with added honey classified as NOVA-4, presented the highest content of acrylamide and HMF.

**Figure 3:**
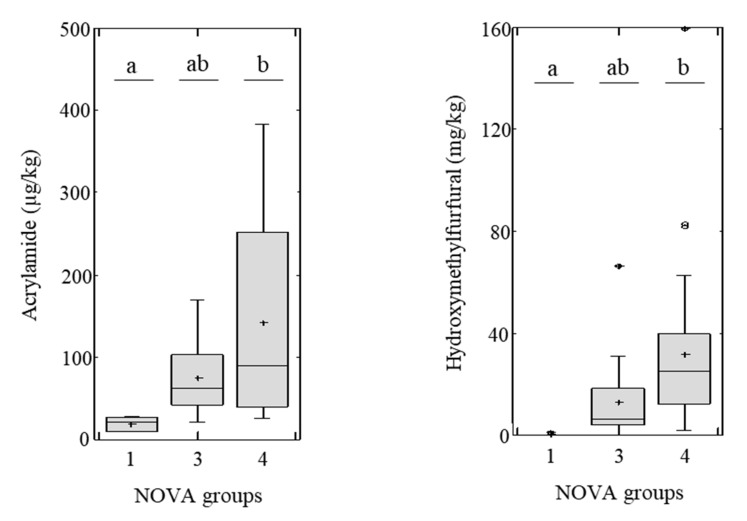
Bar plot showing the distribution of acrylamide (µg/kg) and hydroxymethylfurfural (mg/kg) content across different NOVA groups. Different letters indicate significant differences between NOVA groups (ANOVA and Bonferroni post-hoc test, *p* < 0.05). NOVA-1 (unprocessed or minimally processed foods, *n* = 6), NOVA-3 (processed foods, *n* = 16), NOVA-4 (ultra-processed foods, *n* = 31).

Since there was a strong correlation between the NOVA system, and acrylamide (*p* = 0.0009, *ρ* = 0.4604) and HMF (*p* < 0.0001, *ρ* = 0.5925) (Table 3), further analyses were focused on identifying differences according to NOVA groups. Figure 3 shows acrylamide and HMF distribution within each NOVA group, alongside the resultant significance level. Post-hoc ANOVA tests were applied to explore the differences between multiple groups. Significant differences between NOVA groups were found for acrylamide (*p* = 0.0072) and HMF (*p* = 0.0114). The average acrylamide content of breakfast cereals was 20, 75 and 142 µg/kg for samples classified as belonging to the NOVA-1, NOVA-3 and NOVA-4 groups, respectively (Table 2). Similarly, average HMF content of breakfast cereals was 0.5, 13.3, and 32.1 mg/kg for samples classified as NOVA-1, NOVA-3, and NOVA-4, respectively (Table 2). Although there is almost a two-fold difference in average acrylamide and HMF content between NOVA-3 and NOVA-4 groups, significant differences were not found. Based on the fact that the occurrence of heat-induced chemical markers is not significantly different between processed and ultra-processed breakfast cereals, the application of the NOVA system to breakfast cereals may not reflect the intensity of the thermal treatment. This conclusion is in line with one of the major criticisms of the NOVA system, since the very broad definition of the NOVA classification of food makes difficult to clearly identify the extent of processing [62,63].

**Figure 4:**
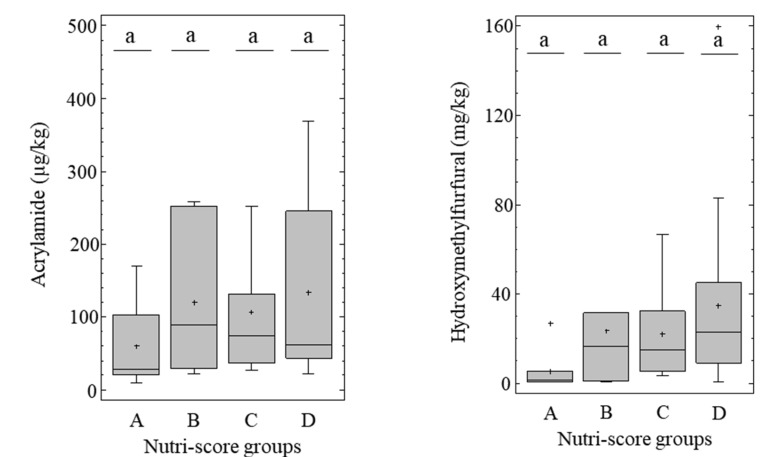
Bar plot showing the distribution of acrylamide (µg/kg) and hydroxymethylfurfural (mg/kg) content between the different NutriScore groups (ANOVA and Bonferroni post-hoc test, *p* < 0.05). Different letters indicate significant differences among Nutriscore groups. Nutri-score A (*n* = 10), B (*n* = 7), C (*n* = 20), D (*n* = 16).

Although the degree of processing is not considered by the NutriScore system, the relationship of NutriScore groups with acrylamide and HMF content was evaluated. Differences between NutriScore groups and acrylamide (*p* = 0.3679) and HMF (*p* = 0.0933) were not statistically significant (*p* < 0.05) (Figure 4). However, there is a clear trend in relation to HMF, as already highlighted through correlational analysis (Table 3). A six-fold difference was seen between the average HMF content of the NutriScore A (5.4 mg/kg) and D (34.6 mg/kg) groups. In the case of acrylamide, average contents are 60 and 133 µg/kg in NutriScore A and D groups, respectively.

Considering children as a segment of the population with high breakfast cereal consumption, greater insight was achieved by focusing on those breakfast cereals especially targeted towards children. No significant differences relating to acrylamide (*p* = 0.0618) and HMF (*p* = 0.0974) were found when comparing the subgroup of samples tailored towards consumption in children (*n* = 17) with those for the general population (*n* = 36). However, we must highlight that average acrylamide content was higher in samples aimed towards children (146 µg/kg, 72–221 µg/kg at 95% C.I.) than for the general population (90 µg/kg, 65–114 µg/kg at 95% C.I.). Similar results were observed for HMF, showing values of 32.4 (13–51.9 mg/kg at 95% C.I.) and 18.3 mg/kg (10.6–20 mg/kg at 95% C.I.) for breakfast cereals intended for children and the general population, respectively.

### 3.5. Strengths and Limitations

The present study presents a number of limitations that require further discussion. In the case of acrylamide, asparagine is the limiting precursor leading to its formation in cereal-derived products. However, protein and amino acid composition differs between cereals and varieties and, therefore, in the absence of other ingredients, the type of cereal used in the formulation of breakfast cereals will influence acrylamide formation [64]. Four main breakfast cereal formulations were identified in the NOVA-4 group, these being oat, maize, wheat and a mixture of various cereals. Wheat and rye have a significantly higher asparagine content than maize and rice. The average asparagine content in rye, wheat, oat, maize, and rice flour was 634, 172, 250–400g, 50–70, and 1–6 mg/kg, respectively [59]. In addition, the content of free asparagine in cereal also depends on the extent of refining and the use of wholegrains [65]. Another source of variation in the NOVA classification for acrylamide is the technology used for breakfast cereal manufacture, as this determines heat input and mass transfer activity. The literature states that extrusion cooking methods with a higher thermal input during direct-expansion extrusion give rise to high acrylamide levels when compared with pellet-to-flaking extrusion [66]. Another important source of variability is the application of a mitigation strategy for acrylamide during the manufacture of breakfast cereals, such as the use of asparaginase in the pre-treatment of dough. In the case of HMF, fewer limitations were identified. HMF is a well-known heat-induced chemical index of the extent of non-enzymatic browning reactions in foods, including caramelization and the Maillard reaction. Sugar type and content, temperature and processing time, moisture and pH will drive the extent of HMF formation [26]. Thus, HMF dependence on sugar content is greater than that seen with acrylamide and, consequently, it has a stronger relationship with NOVA (Table 3). In contrast to acrylamide, HMF formation did not greatly depend on the type of cereal or degree of refining, and any mitigation strategy is required today. In the present study, the main limitation in relation to HMF is the use of ingredients containing HMF or its precursors in the formula which cannot be properly quantified. For both compounds, there is no precise thermal history (temperature/time) of the intensity of the treatment applied, not only for the product recipe itself but also for the extent of processing applied on certain ingredients (syrups, and food extracts). Finally, another limitation of the present study is the lack of prior investigations into the relationship between chemical markers of thermal processing and food classification systems.

Growing evidence indicates a relationship between the consumption of ultra-processed food and the higher prevalence of metabolic diseases (obesity, lipid dysregulation and metabolic syndrome), increased incidence of diabetes type 2, cardiovascular disease and cancer [2,8,67], and higher mortality in the general population [68]. The availability and consumption of ultra-processed foods in Spanish households has increased greatly, as in many other countries. This amounts to an increase in daily energy intake from 11% in the 1990s to 32% in the present day, alongside increases in foods with added sugar content [49]. The NOVA system is globally recognized, particularly in relation to the concept of ultra-processed food, as an effective approach for identifying foods that contribute to increased risks of some chronic diseases, poor nutritional density and higher sugar intake [11,14]. Epidemiologists have not excluded the contribution of process contaminants to consequences of the overconsumption of ultra-processed foods. However, our results fail to confirm that ultra-processed breakfast cereals have significantly higher acrylamide content relative to processed cereals, despite clears trends emerging. To the best of our knowledge, this is the first study to link food classification systems (NutriScore, NOVA systems) and the chemical markers of thermal processing.

## 4. Conclusions

This investigation shows that there are no significant differences in acrylamide and HMF content between the categories of processed (NOVA-3) and ultra-processed (NOVA-4) breakfast cereals. Differences were only significant between the groups of un-/minimally processed (NOVA-1) and ultra-processed breakfast cereals (NOVA-4). Based on the application of HMF as a classical heat-induced chemical marker of the extent of thermal processing in cereals, within the framework of the 53 tested products, the NOVA classification may not reflect the extent of the thermal treatment applied to the product, as opposed to the type and amount of ingredients incorporated. This has to be confirmed with a higher number of products, and from other categories. The classification of a breakfast cereal as ultra-processed does not predict a significantly higher acrylamide and HMF content relative to processed breakfast cereal. However, trends towards higher acrylamide and HMF content are observed, amounting to a change of 75 µg/kg to 13.3 mg/kg in processed breakfast cereals, and 142 µg/kg to 32.1 mg/kg, in ultra-processed breakfast cereals, respectively. Additionally, it is noteworthy that all samples with greater acrylamide content than the benchmark level established by the EU Regulation for each breakfast cereals category are ultra-processed. In addition, acrylamide and HMF content are not statistically different between NutriScore groups, although there is an overall correlation with HMF.

The present investigation confirms that sugar content is the only nutritional descriptor to positively correlate with acrylamide and HMF content in breakfast cereals, regardless of the type of grain and complexity in the formulation. Ingredient composition complexity is the only factor to differentiate processed and ultra-processed breakfast cereals in terms of the occurrence of heat-induced chemical markers of processing. On the other hand, in consideration of the tendency towards a higher acrylamide content in ultra-processed breakfast cereals and the fact that samples with acrylamide content greater than the benchmark level were all ultra-processed breakfast cereals, our results urge a reformulation of the ultra-processed breakfast cereal group. The formulation of ultra-processed breakfast cereals should be balanced with the intensity of thermal treatment applied, avoiding the combination of recipes with high sugar content and intense thermal treatment. It is necessary for policymakers and the industrial breakfast cereal sector to take a stronger focus on the strategies which limit the addition of sugar. This will not only maintain a healthy nutritional profile but also reduce levels of the heat-induced process contaminant.

## Figures and Tables

**Table 1:** NutriScore distribution of unfavorable (energy density, sugar, saturated fatty acids, sodium) and favorable (fiber and protein) descriptors and heat-induced chemical index (acrylamide, hydroxymethylfurfural) content in breakfast cereals (*n* = 53). Confidence interval (C.I.), minimum (min.), maximum (max.).

	Mean	C.I. (95%)	Min.	Max.	Median
**Nutritional profile**					
Energy (kJ/100 g)	1566	(1535–1596)	1322	1946	1569
Sugar (g/100 g)	17.3	(13.5–21.0)	0.7	49.0	17.5
Saturated fatty acids (g/100 g)	0.9	(0.45–1.30)	0.1	9.5	0.6
Sodium (mg/100 g)	277	(206–348)	0	1100	200
Fiber (g/100 g)	8.2	(5.9–10.4)	0.0	33.1	5.0
Protein (g/100 g)	9.3	(8.4–10.2)	4.5	19	8.5
**Heat-induced chemical indexes**					
Acrylamide (µg/kg)	108	(79–137)	10	382	69
Hydroxymethylfurfural (mg/kg)	22.8	(14.9–30.8)	0.3	159.6	13.5

**Table 2:** NutriScore distribution of unfavorable (energy density, sugar, saturated fatty acids and sodium) and favorable (fiber and protein) descriptors and heat-induced chemical indexes (acrylamide and hydroxymethylfurfural) content in breakfast cereals (*n* = 53) according to the NOVA food classification system. Confidence interval (C.I.), minimum (min.), maximum (max.). NOVA-1 (unprocessed or minimally processed foods, *n* = 6), NOVA-3 (processed foods, *n* = 16), NOVA-4 (ultra-processed foods, *n* = 31).

NOVA Group 1	Mean	C.I. (95%)	Min	Max	Median
***Nutritional profile***					
Energy (kJ/100 g)	1511	(1450–1567)	1460	1582	1496
Sugar (g/100 g)	1.3	(0.8–1.8)	0.7	2.0	1.2
Saturated fatty acids (g/100 g)	1.1	(0.8–1.4)	0.6	1,4	1.2
Sodium (mg/100 g)	17	(0–26)	0	100	50
Fiber (g/100 g)	10.7	(8.5–12.9)	9.1	14.7	10.1
Protein (g/100 g)	12.8	(11.1–14.5)	10.5	14.7	13
***Heat-induced chemical indexes***					
Acrylamide (µg/kg)	20	(11–28)	10	28	21
Hydroxymethylfurfural (mg/kg)	0.5	(0.1–1.0)	0.3	1.3	0.3
**NOVA group 3**	**mean**	**C.I. (95%)**	**min**	**max**	**median**
***Nutritional profile***					
Energy (kJ/100 g)	1555	(1500–1610)	1393	1841	1567
Sugar (g/100 g)	11.6	(6.1–17.2)	0.8	37.0	8.0
Saturated fatty acids (g/100 g)	0.8	(0.1–1.4)	0.1	5.2	0.5
Sodium (mg/100 g)	318	(173–463)	0	1080	370
Fiber (g/100 g)	7.6	(2.7–12.3)	0.0	31.0	4.1
Protein (g/100 g)	8.9	(7.4–10.4)	4.5	15.0	8.8
***Heat-induced chemical indexes***					
Acrylamide (µg/kg)	75	(51–99)	22	170	62.5
Hydroxymethylfurfural (mg/kg)	13.3	(4.1–22.4)	0.3	66.3	6.4
**NOVA group 4**	**mean**	**C.I. (95%)**	**min**	**max**	**median**
***Nutritional profile***					
Energy (kJ/100 g)	1582	(1537–1626)	1322	1946	1590
Sugar (g/100 g)	23.1	(18.4–27.7)	1.3	49.0	23.0
Saturated fatty acids (g/100 g)	0.9	(0.3–1.5)	0.1	9.5	0.4
Sodium (mg/100 g)	308	(222–395)	0	880	272
Fiber (g/100 g)	8.0	(4.8–11.2)	0.0	33.1	5.0
Protein (g/100 g)	8.9	(7.6–10.1)	4.7	19.0	8.0
***Heat-induced chemical indexes***					
Acrylamide (µg/kg)	142	(98–187)	26	382	89
Hydroxymethylfurfural (mg/kg)	32.1	(20.1–44.0)	1.9	159.6	25.4

**Table 3:** Bivariate correlations between acrylamide (µg/kg) and hydroxymethylfurfural (HMF, mg/kg) content, NutriScore nutritional descriptors (Pearson coefficient, r), and rank classifications for NOVA and NutriScore groups (Spearman coefficient, *ρ*). Significant values are expressed as *p* < 0.05, ** *p* < 0.01, *** *p* < 0.001, **** *p* < 0.0001. Energy (kJ/100 g), sugar (g/100 g), saturated fat (g/100 g), sodium (mg/100 g), fiber (g/100 g) and protein (g/100 g).

	Acrylamide			Hydroxymethylfurfural		
*Analytical factors*	*r*	*p*		*r*	*p*	
HMF	0.4987	*0.0001*	***			
Energy	0.2369	*0.0876*		0.1399	*0.3179*	
Sugar	0.4648	*0.0005*	***	0.5547	*0.0000*	****
Saturated fat	−0.0020	*0.9885*		0.0910	*0.5260*	
Sodium	−0.1642	*0.2399*		−0.1100	*0.4330*	
Fiber	−0.8220	*0.5583*		0.0082	*0.9536*	
Protein	−0.1573	*0.2606*		−0.1744	*0.2118*	
*Classification factors*	*ρ*	*p*		*ρ*	*p*	
NOVA	0.4604	*0.0009*	***	0.5925	*0.0000*	****
NutriScore	0.2112	*0.1278*		0.3983	*0.0041*	**

## Supplementary Materials

The following are available online at https://www.mdpi.com/2072-6643/12/5/1418/s1, Figure S1: Distribution of breakfast cereals segmented by the largest type of grain (rice, oat, maize, wheat, mixture) according to NOVA classification groups. Numbers are percentage of cases, Figure S2: Contribution of NutriScore descriptors (energy, sugar, saturated fat, sodium, fiber and protein), and heat-induced chemical indexes (acrylamide and HMF) to the mean values of the whole group of breakfast cereals (*n* = 53) across the NOVA categories. NOVA-1 (white circle), NOVA-3 (grey circle), NOVA-4 (black circle). HMF (hydroxymethylfurfural). Lines represent scale at 50%, 100% (dotted line), and 150% from the mean value for each descriptor, Figure S3: Classification of breakfast cereals according to the food group “breakfast cereal” as established by the EU Regulation 2017/2158 on acrylamide mitigation and their distribution across the NOVA system. EU-Group 1 (bran products and whole grain cereals, gun puffed grain), EU-Group 2 (wheat and rye based products), EU-Group 3 (maize, oat, spelt, barley and rice based products). Numbers in percentage are cases, Table S1: NutriScore distribution of the nutrient descriptors (energy density, sugar, saturated fatty acids, sodium, fiber and protein) content in breakfast cereals (*n* = 53) according to the NutriScore classification system. Confidence interval (C.I.), minimum (min.), maximum (max.).
