# Supplementary material for: Association between Heat-Induced Chemical Markers and Ultra-Processed Foods: A Case Study on Breakfast Cereals
Source: Nutrients. 2020 May 14;12(5):1418. doi: 10.3390/nu12051418 (PMC7285198; doi:10.3390/nu12051418)
Supplement: Supplementary file 1 [file nutrients-12-01418-s001.pdf]

Supplementary Material

Association between heat-induced chemical makers and ultra-processed foods: A case study on breakfast cereals

Francisco J. Morales [1,*], Marta Mesías [1] and Cristina Delgado-Andrade [1]

Figure S1. Distribution of breakfast cereals segmented by the largest type of grain (rice, oat, maize, wheat, mixture) according to NOVA classification groups. Numbers are percentage of cases.

.

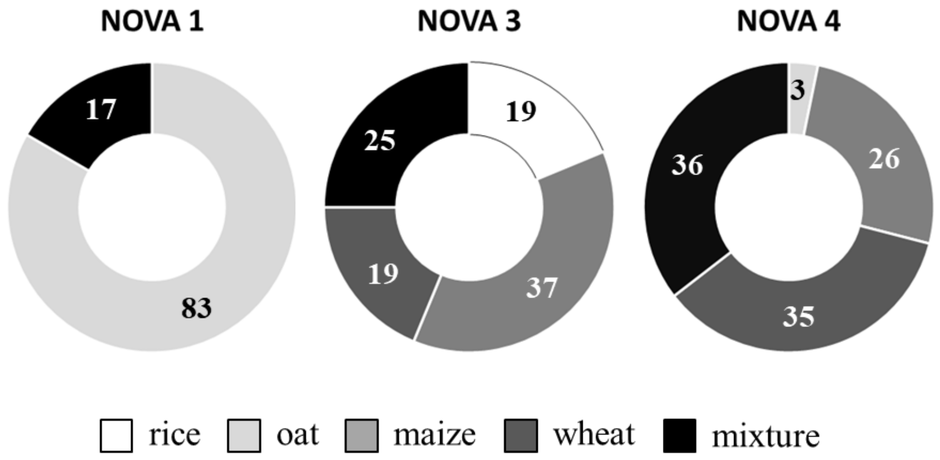

Supplementary Material

Association between heat-induced chemical makers and ultra-processed foods: A case study on breakfast cereals

Francisco J. Morales [1,*], Marta Mesías [1] and Cristina Delgado-Andrade [1]

Figure S2. Contribution of NutriScore descriptors (energy, sugar, saturated fat, sodium, fiber and protein) and heat-induced chemical indexes (acrylamide and HMF) to the mean values of the whole group of breakfast cereals (*n* = 53) across the NOVA categories. NOVA-1 (white circle), NOVA-3 (grey circle), NOVA-4 (black circle). HMF (hydroxymethylfurfural). Lines represent scale at 50%, 100% (dotted line), and 150% from the mean value for each descriptor.

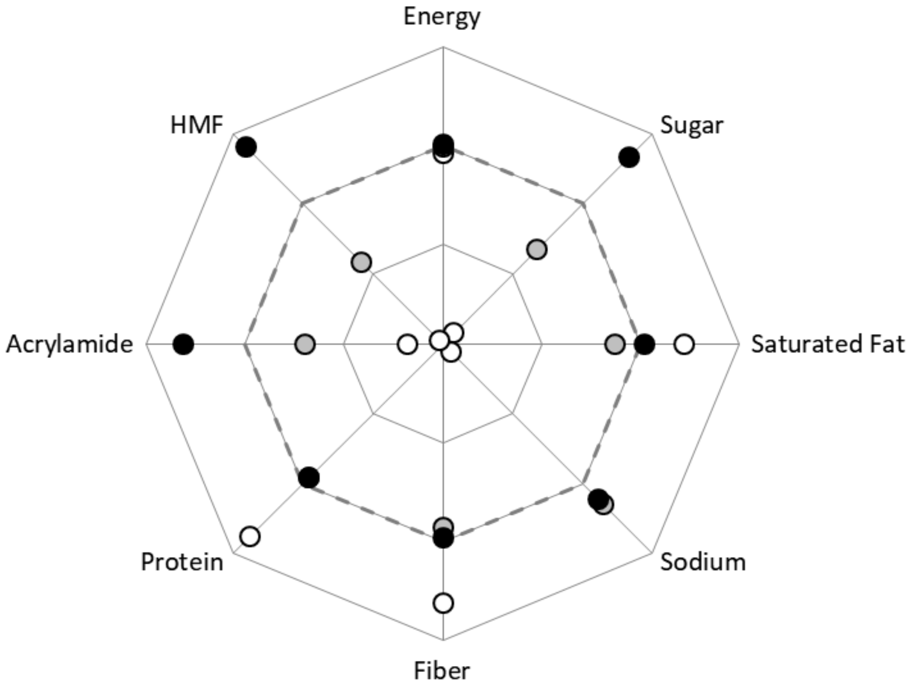

Supplementary Material

Association between heat-induced chemical makers and ultra-processed foods: A case study on breakfast cereals

Francisco J. Morales [1,*], Marta Mesías [1] and Cristina Delgado-Andrade [1]

Figure S3. Classification of breakfast cereals according to the food group "breakfast cereal" as established by the EU Regulation 2017/2158 on acrylamide mitigation and their distribution across the NOVA system. EU-Group 1(bran products and whole grain cereals, gun puffed grain), EU-Group 2 (wheat and rye based products), EU-Group 3 (maize, oat, spelt, barley and rice based products). Numbers in percentage are cases.

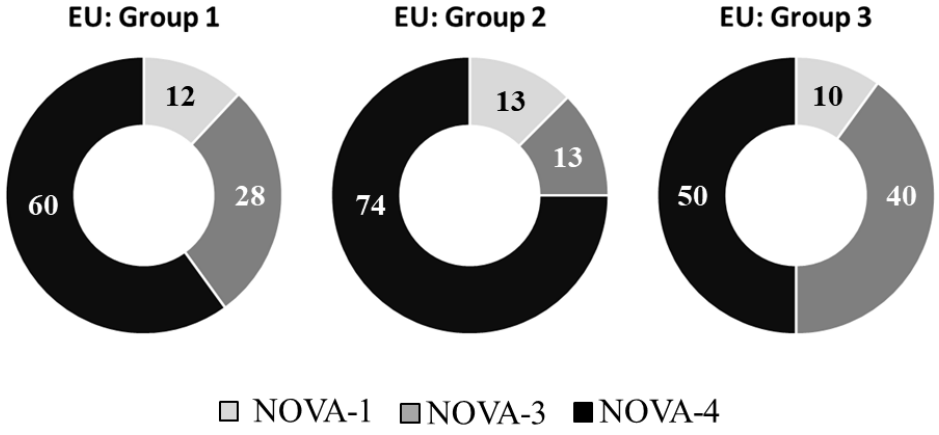

Supplementary Material

Association between heat-induced chemical makers and ultra-processed foods: A case study on breakfast cereals

Francisco J. Morales [1,*], Marta Mesías [1] and Cristina Delgado-Andrade [1]

Table S1. NutriScore distribution of the nutrient descriptors (energy density, sugar, saturated fatty acids, sodium, fiber and protein) content in breakfast cereals (*n* = 53) according to the NutriScore classification system. Confidence interval (C.I.), minimum (min.), maximum (max.).

**NutriScore A** *(n = 10)*	**mean**	**C.I.(95%)**	**min.**	**max.**	**median**
Energy (kJ/100 g)	1524	(1488 - 1560)	1460	1615	1513
Sugar (g/100 g)	1.9	(0.7 – 3.1)	0.7	4.8	1.2
Saturated fatty acids (g/100 g)	0.80	(0.53 – 1.07)	0.20	1.30	0.75
Sodium (mg/100 g)	80	(0 - 168)	0	400	50
Fiber (g/100 g)	8.6	(6.1 – 11.2)	3.3	14.7	9.8
Protein (g/100 g)	12.0	(9.5 – 14.6)	7.3	19.0	11.8

**NutriScore B** *(n = 7)*	**mean**	**C.I.(95%)**	**min.**	**max.**	**median**
Energy (kJ/100 g)	1475	(1382 - 1569)	1331	1582	1502
Sugar (g/100 g)	12.5	(6.2 – 18.9)	2	23.0	13
Saturated fatty acids (g/100 g)	0.70	(0.36 – 1.03)	0.20	1.40	0.70
Sodium (mg/100 g)	271	(133 - 410)	50	400	300
Fiber (g/100 g)	18.9	(9.2 – 28.6)	3.0	31.0	19.0
Protein (g/100 g)	12.2	(9.7 – 16.6)	7.0	15.0	12.0

**NutriScore C** *(n = 20)*	**mean**	**C.I.(95%)**	**min.**	**max.**	**median**
Energy (kJ/100 g)	1572	(1520 - 1625)	1322	1841	1578
Sugar (g/100 g)	19.6	(14.2 – 25.0)	1.3	43.0	20.2
Saturated fatty acids (g/100 g)	0.69	(0.16 – 1.22)	0.10	5.20	0.30
Sodium (mg/100 g)	305	(208 - 401)	0	700	350
Fiber (g/100 g)	6.8	(2.7 – 11.1)	0.0	33.1	4.9
Protein (g/100 g)	6.9	(7.0 – 9.4)	5.6	15.3	7.6

**NutriScore D** *(n = 16)*	**mean**	**C.I.(95%)**	**min.**	**max.**	**median**
Energy (kJ/100 g)	1623	(1561 - 1684)	1431	1946	1594
Sugar (g/100 g)	25.0	(17.4 – 32.7)	2.7	49.0	24.4
Saturated fatty acids (g/100 g)	1.28	(0.02 – 2.53)	0.10	9.50	0.60
Sodium (mg/100 g)	369	(183 - 554)	0	1100	350
Fiber (g/100 g)	4.8	(3.0 – 6.5)	0.0	12.0	3.8
Protein (g/100 g)	7.8	(6.5 – 9.0)	4.5	13.0	8.1
